# Complex intervention modelling should capture the dynamics of adaptation

**DOI:** 10.1186/s12874-016-0149-8

**Published:** 2016-05-04

**Authors:** James Greenwood-Lee, Penelope Hawe, Alberto Nettel-Aguirre, Alan Shiell, Deborah A. Marshall

**Affiliations:** Centre for Science, Athabasca University, Athabasca, T9S 3A3 Canada; Menzies Centre for Health Policy and The Australian Prevention Partnership Centre, University of Sydney, Sydney, NSW 2006 Australia; Departments of Paediatrics and Community Health Sciences, University of Calgary, Alberta Children’s Hospital Research Institute, Calgary, T3B 6A8 Canada; School of Psychology and Public Health, La Trobe University and The Australian Prevention Partnership Centre, Melbourne, 3086 Australia; Department of Community Health Sciences, University of Calgary, Calgary, T2N 4Z6 Canada

**Keywords:** Complex intervention, Complex adaptive systems, Modeling, Non-linear dynamics, Intervention studies

## Abstract

**Background:**

Complexity has been linked to health interventions in two ways: first as a property of the intervention, and secondly as a property of the system into which the intervention is implemented. The former recognizes that interventions may consist of multiple components that act both independently and interdependently, making it difficult to identify the components or combinations of components (and their contexts) that are important mechanisms of change. The latter recognizes that interventions are implemented in complex adaptive systems comprised of intelligent agents who modify their behaviour (including any actions required to implement the intervention) in an effort to improve outcomes relative to their own perspective and objectives. Although an intervention may be intended to take a particular form, its implementation and impact within the system may deviate from its original intentions as a result of adaptation. Complexity highlights the challenge in developing interventions as effective health solutions. The UK Medical Research Council provides guidelines on the development and evaluation of complex interventions. While mathematical modelling is included in the guidelines, there is potential for mathematical modeling to play a greater role.

**Discussion:**

The dynamic non-linear nature of complex adaptive systems makes mathematical modelling crucial. However, the tendency is for models of interventions to limit focus on the ecology of the system - the ‘real-time’ operation of the system and impacts of the intervention. These models are deficient by not modelling the way the system reacts to the intervention via agent adaptation. Complex intervention modelling needs to capture the consequences of adaptation through the inclusion of an evolutionary dynamic to describe the long-term emergent outcomes that result as agents respond to the ecological changes introduced by intervention in an effort to produce better outcomes for themselves. Mathematical approaches such as those found in economics in evolutionary game theory and mechanism design can inform the design and evaluation of health interventions. As an illustration, the introduction of a central screening clinic is modeled as an example of a health services delivery intervention.

**Summary:**

Complexity necessitates a greater role for mathematical models, especially those that capture the dynamics of human actions and interactions.

**Electronic supplementary material:**

The online version of this article (doi:10.1186/s12874-016-0149-8) contains supplementary material, which is available to authorized users.

## Background

Health researchers have a long history of looking to ecological systems as a metaphor and framework for understanding human endeavours [1–3]. To this end, the concept of “complexity” has been linked to interventions. Here, the meaning of *complex* is not the opposite of *simple*. Rather, it is a loosely defined term used to indicate the importance of relationships and adaptive interactions of parts in the emergence of the whole. The notion of complexity has been linked to interventions in two ways. The first recognizes that interventions can be complex, consisting of multiple components with different targets, which may produce non-linear and difficult to predict effects [4]. The second recognizes that interventions (complex in themselves or not) are embedded within complex adaptive systems (CAS) [5–7]. A CAS is a system comprised of intelligent agents, where each agent has a set of objectives and attempts to achieve these objectives through a process of adaptation. In this context, adaptation refers to the process of change that results as various intelligent agents - from policy maker to patient - who modify their behaviour (including any actions required to implement the intervention) in an effort to improve outcomes relative to their own perspective and objectives. Thus, although an intervention is designed with one outcome effect in mind, its implementation and impact within the system may deviate from its design as a result of adaptation.

This process of adaptation makes the development and implementation of effective and sustainable interventions difficult, and drives the development of increasingly complex interventions. Indeed, interventions must be adaptable both over time and across a diversity of settings to ensure that the adoption and effectiveness of the intervention is not compromised as the population responds to both the direct and indirect changes introduced by the intervention [8].

The high number of linkages between the components of the intervention and the system in which it operates means that change in long term, system-level outcomes may be non-linear, emergent and difficult to predict. As such, a broader approach is needed to design, evaluate and implement complex interventions. The UK Medical Research Council (MRC) guidance on complex interventions [9, 10] is an authoritative, frequently cited reference that continues to evolve and shape health research [11, 12]. The guidance places emphasis on theory driven approaches that demonstrate causal mechanisms between the intervention and its outcomes [13]. While the MRC guidance highlights the value of modelling, it could go much further. To reach its full potential, we argue that mathematical modelling must be embraced within intervention research, but to do so modelling approaches must be expanded. In particular, mathematical modelling in intervention research must endeavor to capture the evolutionary dynamics of adaptation, which are foundational to the study of complexity.

## Discussion

### Complexity necessitates mathematical modelling

Complexity requires scientific approaches with a commitment to holism and interconnectedness. The MRC guidelines recommend the use of *logic models* to describe the theory of the intervention. In this context, a logic model describes the causal pathway by detailing the operation of intervention, the expected effects of its constituent parts, the functions they fulfill (alone and interactively in permutation), and the mechanisms that produce these effects [4]. Logic models certainly play an important role in capturing the theory of the intervention [14, 15]. However, no matter how sophisticated, a logic model alone is not sufficient, as complexity cannot be understood purely through qualitative description; hence tools such as mathematical models, are needed to test the theory of the intervention.

A mathematical model can be constructed as a representation of the logic model, using mathematics to describe the relationships depicted therein and to provide the necessary tools to analyze the model while maintaining rigorous logic. Mathematical models provide greater theoretical precision than models specified in non-mathematical terms in that their underlying assumptions are more easily examined, they are more easily verified (or falsified), and they lend themselves more readily to data analysis through the generation of predictions that can be tested through observation and experimental manipulation [16–18]. Of course, mathematical models are far from infallible. A mathematical model is only as good as our understanding of the system, but in this sense, they are tools for systematically and rigorously exploring the theory of intervention; used to identify research questions that will lead towards the validation of the intervention’s theory or expose its failures, generating predictions as to how the system should respond under experimentation, and driving the collection of data needed for evaluation [19] to assess the effectiveness of the delivered intervention but also its implementation, including aspects of fidelity, dose and reach [12].

### The importance of the dynamics of adaptation

The purpose of modelling is to demonstrate the theory behind the intervention in order to:

1. Highlight why the system is failing (and requires intervention), and;
2. Identify and demonstrate mechanisms through which the system can be influenced via intervention. To this end, mathematical models of complex interventions need to describe two dynamic processes: an ecological one and an evolutionary one.

#### Ecological dynamic

The ecological dynamic captures the ‘real-time’ operation of the system and short term impacts of the intervention by detailing, for example, the health status of the population and their seeking of care in relationship to the resources and infrastructure available to provide services to this population, whether those services are preventative population health interventions or medical services delivered through the health care system. Modelling of health systems in these terms is common-place and capturing the ecological dynamics of a complex intervention, thus far, does not require new methods. It simply requires a broader ‘whole systems’ perspective to ensure that both local and system wide effects are captured [20]. Recent articles provide guidelines on various dynamic modelling approaches and their potential applications, highlighting examples in the evaluation of health services delivery interventions [21, 22].

Modelling the ecological dynamic in its current state provides insight into why the system is failing and why intervention is needed, while modelling the ecological dynamic in a ‘designed’ or ‘optimized’ state provides insight as to potential intervention strategies that can be used to improve the system. However, caution is required. Models that only consider the ecological dynamics of the system are deficient by not modelling the way the system reacts to the intervention via agent adaptation; effectively ignoring agent driven adaptation as both an explanation for why the system is failing, and as an mechanism of change that influences the realities of implementation and sustainability of the intervention and its long term effects.

#### Evolutionary dynamic

The evolutionary dynamic describes the process of adaptation that creates change in the ecological dynamic. It is a description of long term emergent outcomes that result from a utility driven feedback mechanism, in which operational processes and strategies are evaluated as agents seek to produce better outcomes for themselves. This includes how agents evaluate and respond to the short-term impacts of the intervention, adapting, and adopting or discarding the intervention according to how it meets their interests and needs.

Mathematical models that incorporate adaptive processes are important to our understanding of complex interventions but such models are not typically employed in intervention development. The modelling itself is not the primary challenge. Mathematical approaches such as those found in economics in evolutionary game theory and mechanism design (see [23, 24] for examples of applications in health settings) can inform the design and evaluation of health interventions. In these approaches, the strategic interactions that occur as agents evaluate and re-evaluate the different strategies available to them are modelled as a dynamic game in which one group (the group intervening) may exert a certain amount of influence over the other groups. Within the game, the intervening group is tasked with designing the rules by which the others must play, with the objective of achieving a specific outcome that is of system-wide benefit, even though each agent group has its own set of objectives. Within this framework the intervention is represented as a strategy employed by the agent group implementing the intervention to steer the system to a desired state (e.g. [25–27]), but the response of recipient agent groups is equally important in determining whether the intervention is successful.

Conflicting interests among different agent groups can make it difficult to implement and sustain an intervention. Such conflict may arise simply as a matter of perspective; trade-offs deemed acceptable by policy makers may not align with the perspective of care providers, where the former take a broad view of the system’s operation, while the latter take a view that is focused on the patients in their care. Methods such as multi-criteria decision analysis [28, 29] can be used to measure the differing priorities of stakeholder groups in decision making processes (e.g. [30]) enabling the perspectives of different stakeholder groups to be captured within mathematical models. As such, conflict can be identified and its implications identified as risks to the intervention, allowing the intervention to be re-designed, perhaps to include formative feedback processes that are designed to ease conflict.

Agent based modelling approaches like those used in mechanism design should resonate with complex interventions. The task of those involved in intervention development is to align objectives by creating incentives and opportunities for individuals and organizations to alter and align their behaviours and operations in a manner that achieves a desired outcome for the recipients *and* those implementing and/or designing the intervention. That is, the art of intervention development is in creating the conditions that encourage stakeholder behaviour that benefits the health of the population as a whole, recognising the multiple objectives, time horizons, decisions and decision makers. It seems that the primary challenge to the development of mathematical models of complex interventions that incorporate adaptive processes lies in acknowledging the need for the inclusion of evolutionary dynamics. To this end guidance on complex intervention modelling, like those on the development [9, 10] and evaluation [11, 12] of complex interventions is needed.

### An example – referral to next available specialist via centralized referral clinics

To illustrate the importance of a system’s evolutionary dynamics, we model the introduction of a “next available specialist” referral option via a central screening clinic as an example of a health services delivery intervention. We describe the model and the outcomes of our analysis below; the details of the model and its mathematical analysis are presented in the Additional file 1.

#### The intervention – Referral to next available specialist via a central screening clinic

Central intake clinics have been proposed as a mechanism to improve the primary-specialty care interface by ensuring patients receive appropriate services in a timely manner by screening patients and subsequently scheduling consults to the next available specialist. In this example we consider a scenario in which all referrals are pooled and distributed amongst a network of *n* specialists. However, two options are presented for the routing of the referral. In the first option, referrals are sent to a centralized screening clinic, and in the second option the referral is sent directly to the next available specialist. In both cases, the patient will undergo screening prior to the specialist consult. Each specialist can accept referrals from the central screening clinic, or direct referrals, or both. The specialist simply allocates a proportion of available consults to the central screening clinic for scheduling, leaving the remainder for direct referrals. Our purpose is to assess whether the central screening clinic is effective and sustainable under this scenario. For ease of presentation, the model is intentionally simple. A key assumption of our model is that specialists are identical in their capacity to see patients. Clearly this is not true, but practically speaking this ensures the model is analytically tractable, which makes the presentation of its analysis straight forward. However, in its simplicity the example highlights that the intervention and the system it operates in are influenced by adaptive dynamics without being complicated.

#### The ecological dynamic

To understand how the centralized screening clinic could improve wait times we can model the ecological dynamics of the system. Indeed, such models have shown that the centralized intake of referrals and subsequent scheduling to the next available specialist improves wait times from referral to specialist consult [31, 32]. In essence, excessive wait times are caused by local imbalances in supply and demand, whether the cause be stochastic or deterministic in nature.

**Fig. 1:**
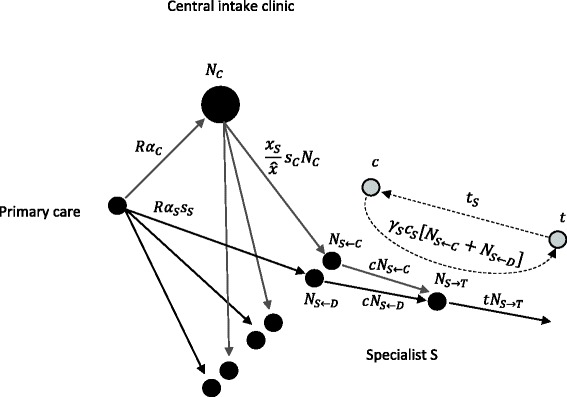
Depiction of referrals from primary to specialist care under a hybrid direct referral/centralized intake system. Black dots indicate queues of patients. Gray arrows indicate patient flows routed through the central intake clinic. Black arrows depict patient flows from primary care direct to specialty care. Gray dots and dotted arrows depict the engagement cycle of specialist resources. The change in the number of patients waiting in given queue per unit time is equal to the flow in minus the flow out. For example: ${\overset{.}{N}}_C=R{\alpha}_C-{\sum}_S\frac{x_S}{\widehat{x}}{s}_C{N}_C=R{\alpha}_C-{s}_C{N}_C$

In the Additional file 1 - Part A, we present a simple model of the ecological dynamics of our example system shown in Fig. 1. Our analysis demonstrates that the proposed introduction of the centralized screening clinic will improve wait times compared to the direct referral system. For this to occur, the central screening clinic must be more efficient than local screening processes. The magnitude of the observed wait time improvements will depend on the level of participation of specialists as described by their allocation of consults to the central intake clinic for scheduling. Little improvement occurs when this allocation is small. In contrast, wait times are minimized when specialists allocate all consults to the central intake clinic for scheduling.

#### Why we need an evolutionary dynamic

Our analysis of the ecological dynamics revealed that system performance is determined by the specialists’ allocation strategies, defined as the proportion of available consults allocated to the central intake clinic for scheduling. Thus, the reaction of the specialists to the introduction of the central intake clinic as they adapt their allocation strategies will have implications on the effectiveness and sustainability of the intervention. If we only consider the ecological dynamics of the system, then our analysis is deficient as it does not consider the way the specialists react to the intervention.

#### The evolutionary dynamic

In the Additional file 1 - Part B, we use a utility driven feedback mechanism to model how specialists react to the introduction of the central intake clinic by considering the adaptation of their allocation strategies over time. Here, we assume that the primary objective of the specialist is to maximize throughput as measured by the number of patients entering treatment per unit time. Mathematically, a game is established as specialist utility is not only determined by the specialist’s allocation strategy, but also by the strategy of the other specialists.

Our analysis of the evolutionary dynamics reveals that each specialists adapts its allocation strategy to create balance between the throughput that is generated from referrals sent by the central screening clinic and throughput that is generated by referrals sent directly to the specialist. Specialists are inclined to allocate a greater proportion of available consults to the central screening clinic for scheduling (at equilibrium) when referrals to the central screening clinic are high. If relatively few patients are referred through the central intake clinic, then specialists are expected to decrease their allocation of consults to the central intake clinic. The long term outcome is that specialist allocations to central intake decline to the point that central screening clinic no longer offers substantial wait time benefit and its continued operation may not be justified. However, if enough patients are referred through the central intake clinic, it becomes in the specialists’ best interest to increase their allocation of available consult appointments to the central intake clinic. If this feedback process is strong enough, then the long term outcome is that specialists allocate 100 % of available appointments to the central screening clinic, and optimal system performance is achieved. The ecology of the system is changed.

#### Beyond the simple model

The example was kept simple for illustrative purposes. However, the model is easily made more complicated by relaxing assumptions and capturing more realistic representations of both ecological and evolutionary dynamics. For example, a key assumption of the model is that specialists are identical in characteristics, receive an equal proportion of direct referrals, effectively treating them as homogenous group. If we relax these assumptions, then each individual specialist will vary their allocation strategies independently and we expect that specialists with established practices are much less inclined to allocate available consult appointments to the central screening clinic as these are easily filled by direct bookings. This may not be the case for specialists with new practices, who will benefit from referrals gained trough the central screening clinic. This may alter the outcomes we reported in the simple example above, especially as they relate to the sustainability of the intervention.

Another complication that should be considered is the adaptive behavior of patients/referring physicians. In our analysis, we observed that the specialists’ allocation strategies evolved in response to the proportion of referrals sent to the central intake clinic; a decision that is made by the patient/referring physician. Thus, the model presented here presents an incomplete picture of the evolutionary dynamics. Rather, the co-evolution of the adaptive behaviours of the patient/referring physician and the specialists should be considered. Two extreme scenarios present themselves. In the first, patients perceive benefit from the central screening clinic option and prefer it over direct referrals. This preference of patients entices specialists to allocate a higher proportion of available consults to the central screening clinic. This creates a positive feedback loop and the predicted long term outcome is full participation in the central intake clinic. In the second scenario, a positive feedback loop is again present, but with opposite outcome. Patient’s initially perceive little value in the central screening clinic option, and show little preference for the option. This lack of preference causes specialists to decrease their allocation of available consults to the central screening clinic, which further decreases patient/referring physician preference for the next available specialist option. Further model development provides a tool to explore the conditions that lead to each scenario.

## Conclusion

Complexity brings forth a natural role for mathematical modelling to verify and test the theory of the intervention from the earliest stages of development through to its evaluation. To reach its full potential, mathematical modelling of complex interventions must capture the dynamics of adaptation to demonstrate causal mechanisms while considering the implementation and sustainability of the intervention. Intervention research must harness the adaptation process to create interventions that better fit the diversity of settings into which they are implemented in the expectation that this will enhance the effectiveness and sustainability of efforts to improve health.

### Ethics approval and consent to participate

Not Applicable.

### Consent for publication

Not Applicable.

### Availability of data and materials

No data was used. Mathematical details of the model are presented in the Appendix, included as an additional file.

## Additional file

Additional file 1: Model details - referral to next available specialist via centralized screening clinics. (PDF 430 kb)

## Abbreviations

CAS: complex adaptive system
MRC: The UK Medical Research Council
