# Supplementary material for: Complex intervention modelling should capture the dynamics of adaptation
Source: BMC Med Res Methodol. 2016 May 4;16:51. doi: 10.1186/s12874-016-0149-8 (PMC4855763; doi:10.1186/s12874-016-0149-8)
Supplement: Additional file 1: — Model details - referral to next available specialist via centralized screening clinics. (PDF 430 kb) [file 12874_2016_149_MOESM1_ESM.pdf]

Additional file 1: Model details - referral to next available specialist via centralized screening clinics.

Part A. The ecological dynamics

We develop a simple high-level model describing the operation of health system with two options for referring patients from primary to specialty care. The model is presented in Figure 1 (main article) and notation is summarized in Table 1.

Table 1. Model Notation

Notation	Definition
n	Number of specialists.
N_C	Number of patients waiting for screening at the central intake clinic (patients).
$N_{S\leftarrow C}$	Number of patients waiting for consult with specialist, who were referred by central intake clinic (patients).
$N_{S\leftarrow D}$	Number of patients waiting for consult with specialist, who were referred directly to the specialist (patients).
$N_{S\rightarrow T}$	Number of patients waiting for entering treatment with the specialist (patients).
R	Referral rate (patients/time).
α_C	Proportion of referrals sent to the central intake clinic.
α_S	Proportion of referrals sent to specialist S.
s_C, s_D	Screening rate central screening clinic and by specialist respectively (1/time)
c_S	First consult rate (1/time)
t_S	Treatment rate (1/time)
$C_S = c_S + t_S$	Total Consult rate (1/time)
γ_S	Patient to resource conversion rate (1/patient)
A_C	Proportions of patients appropriate for Specialist care, who were referred from the central intake clinic
A_D	Proportions of patients appropriate for Specialist care, who were referred directly to specialist *S*.
x_S	Proportion of available consult appointments that specialist *S* allocates to the central intake clinic for scheduling.
$\hat{x} = \sum_S x_S$	Total allocation of available consult appointments to the central intake clinic.
x	Altered specialist allocation strategy
x^*	Normal specialist allocation strategy

In our model all referrals are pooled and distributed amongst a network of *n* specialists. Under the first option, referrals are sent to a centralized intake clinic for screening (at rate $R\alpha_C$). Once referred to the central intake screening clinic, the patient joins a queue of N_C patients, who await an initial screening appointment (provided at rate $s_c N_C$). To be eligible to receive referrals from the central intake clinic

specialists must allocate a proportion x_S of available consult appointments for scheduling via the central intake screening clinic. The proportion of referrals received is then given by $x_S/\hat{x}$. Patients referred to specialist via the central intake screening clinic are then placed on the specialists wait list, $N_{S\leftarrow C}$, and await consult (provided at rate $c_S N_{S\leftarrow C}$). Thus, the movement of patients from primary care to specialist consult via the central intake clinic is given by:

$$\dot{N}_C = R\alpha_C - sN_C$$

$$\dot{N}_{S\leftarrow C} = \frac{x_S}{\hat{x}} sN_C - c_S N_{S\leftarrow C}$$

Under the second option, referrals are sent directly to a specialist (at rate $R\alpha_S$) for screening and subsequent consult. Specialists allocate a proportion $(1 - x_S)$ of available consult appointments for direct referrals. Patients referred directly to the specialist are then screened at rate s_s and placed on the specialist's wait list, $N_{S\leftarrow D}$, and await consult (provided at rate $c_S N_{S\leftarrow D}$). Thus, the movement of patients from primary care to specialist consult via the direct referrals is given by:

$$\dot{N}_{S\leftarrow D} = R\alpha_s(1 - x_S) - c_S s_s N_{S\leftarrow D}$$

Patients that can be treated by the specialist are added to the specialists practice for treatment, $N_{S\rightarrow T}$. The proportion of patients that receive specialty care is given by A_C and A_D for patients who were screened at the central intake clinic and patients referred directly to specialist respectively. That is, A_C and A_D can be thought of the positive predictive values of the central screening clinic, and specialist screening processes respectively. Treatment is provided at rate $t_S N_{S\rightarrow T}$. Thus, the movement of patients from consult into treatment is given by:

$$\dot{N}_{S\rightarrow T} = c_S(A_C N_{S\leftarrow C} + A_D s_s N_{S\leftarrow D}) - t_s N_{S\rightarrow T}$$

Finally, we note that specialists have limited appointments that must be allocated amongst patients needing first consults, and those needing treatment. Appointments for new consults are freed as treated

patients leave the specialist's care (at rate t_S), and are booked at a rate proportional to the consult rate, $\gamma_S c_S[N_{S\leftarrow C} + s_s N_{S\leftarrow D}]$. The dynamics describing the specialist's appointment availability are:

$$\dot{c}_S = t_S - \gamma c_S[N_{S\leftarrow C} + s_s N_{S\leftarrow D}]$$

$$\dot{t}_S = \gamma c_S[N_{S\leftarrow C} + s_s N_{S\leftarrow D}] - t_S$$

Since, $\dot{c}_S = -\dot{t}_S$, it follows that the number of appointments available is constant, $C = c_S + t_S$.

Analysis of the ecological dynamics

The ecological dynamics of the system are straightforward. If capacity exceeds demand throughout the health service network, $\left(\alpha_S(1-x) + \alpha_c \frac{x}{\hat{x}}\right) < \frac{C}{R\gamma}$ for all specialists, then the system will reach an equilibrium state given by:

$$N_C^* = \frac{R\alpha_c}{s_c}, N_{S\leftarrow C}^* = \frac{x_S}{\hat{x}}\frac{R\alpha_C}{c_S^*} \quad N_{S\leftarrow D}^* = \frac{R\alpha_S(1-x_S)}{s_S c_S^*}, N_{S\rightarrow T}^* = \frac{R(A_D\alpha_S(1-x_S) + A_C\frac{x_S}{\hat{x}}\alpha_C)}{t_S^*}$$

$$c_S^* = C - \gamma R\left(\alpha_c\frac{x_S}{\hat{x}} + \alpha_S(1-x_S)\right)$$

Performance analysis

The ecological dynamics are now analyzed to describe system performance. Here, we measure performance through wait times. The expected wait times from referral to specialist consult for a patient referred via the central intake clinic, W_C, and for a patient referred directly to a specialist, W_D are, respectively:

$$W_C = \frac{1}{s} + \sum_S \frac{x_S}{\hat{x}}\frac{1}{c_S^*}, \text{and} \quad W_D = \frac{1}{s_s} + \frac{1}{c_S^*}.$$

Thus, the expected wait time for patients seeking specialty care is:

$$W = \alpha_c W_c + \sum_S \alpha_s W_D = \alpha_C \left[\frac{1}{s} + \sum_S \frac{x_S}{\hat{x}} \frac{1}{c_S^*}\right] + \sum_S \alpha_S \left[\frac{1}{s_S} + \frac{1}{c_S^*}\right]$$

Here, $\sum_S \alpha_S W_D$ is minimized when referrals are equally distributed amongst specialists, $\alpha_S = \frac{1-\alpha_c}{n}$, which is assumed. Referral via the central intake screening clinic creates additional wait time improvements if the wait time resulting from the central intake screening process, $1/s_c$ is less than the wait time resulting from the screening via a direct referral, $1/s_S$. However, wait time improvements will be dependent on how specialists allocate available consult appointments between referrals received from the central intake clinic and direct referrals as captured in the first consult rate, c_S^*. The optimized state of the system -with the intervention in place- occurs when $\alpha_C = 1$, $\alpha_S = 0$ and $x_S = 1$ for all *S*. That is, the referrals from primary care to specialty care are optimized when all specialists are referred patients via the central intake clinic; the expected wait time is

$$W = W_C = \left[\frac{1}{s} + \sum_S \frac{1}{n} \frac{1}{C - \frac{\gamma R}{n}}\right].$$

Part B. The evolutionary dynamics

We now describe the evolutionary dynamics that create change in the system's ecological dynamic. Specifically, we model how specialists allocate available consult appointments between referrals received from the central intake clinic and direct referrals using a utility driven feedback mechanism. Specifically, we assume that the specialist seeks to maximize throughput so as to treat as many patients per unit time as is possible, where throughput is given by the utility function:

$$U_S = c_S^*[A_C N_{S\leftarrow C}^* + A_D N_{S\leftarrow D}^*] = R\left[A_C \alpha_C \frac{x}{\hat{x}} + A_D \alpha_S (1-x)\right].$$

To examine how a specialist's utility is affected by allocation strategy *x* we can examine the marginal return, which describes the gain in utility awarded when through a small change in the allocation strategy. Here, we must recognize that a game is established, as specialist utility is not only determined by the

specialist's allocation strategy, but also by the strategy of the other specialists. To calculate the marginal return, we consider a focal specialist, and ask how that specialist's payoff is altered by a marginal increase (above the average, x^*) in the allocation of consult appointments to patients choosing next available specialist, $x = x^* + Dx$. The altered payoff to our focal specialist (as a first order approximation) is:

$$\left.\frac{\partial U_S}{\partial x}\right|_{x=x^*} \approx -A_D\alpha_S + \frac{A_C\alpha_C}{nx^*}.$$

In words, a small increase in the allocation of consults to patients choosing next available specialist, results in the gain of throughput acquired through central intake at the expense of throughput lost from direct referrals.

If we assume a small amount of variation among the *x* of our specialist group, normally distributed about a mean x^* then the evolutionary dynamic describing the adaptation process can be written as

$$\dot{x^*} = var(x)\left.\frac{\partial U_S}{\partial x}\right|_{x=x^*}.$$

Which simply states that the change in the mean of x^* is determined as the product of the variation in *x*, and the marginal return produced when using strategy *x*, in a system where the strategy $x^*$is typically used. For this to be true, the change in *x* must be purely driven by the adaptation process. Note the above is a simple form of a replicator dynamic; aptly named because decision strategies that produce higher payoffs are "replicated" and increase in frequency, whereas decision strategies with lower payoffs decrease in frequency.

Analysis of the evolutionary dynamic.

The direction of change observed in x^* is determined by the sign of the marginal return:

$$\left.\frac{\partial U_S}{\partial x}\right|_{x=x^*} \approx -A_D\alpha_S + \frac{A_C\alpha_C}{nx^*}.$$

The challenge of implementation is revealed by examining $\partial U_S/\partial x|_{x=x^*\approx 0}$. If referrals to the central intake clinic are low (i.e. $\alpha_C < x^*$ as $x^* \to 0$), then specialists will prefer to allocate an increased proportion of consults to direct referrals. In such cases, the introduction of the central screening clinic will be difficult if not impossible to implement.

Implementation of the central intake clinic requires that increased participation of specialists be beneficial to them. For this to be achieved the proportion of referrals sent to the central screening clinic at start up, coupled with the benefit offered to the specialist by the screening process, must be sufficiently high, $\frac{A_C}{A_D}\alpha_C > \alpha_S n x^*$. This may be achievable if referrals to the central intake clinic are at least equal to the initial specialist allocations, $\alpha_C \geq x^*$ as $x^* \to 0$.

Assuming that the central intake clinic offers some benefit to specialists, then the system will eventually settle to an evolutionary equilibrium given by:

$$x^* = \frac{A_C \alpha_C}{A_D \alpha_S n}.$$

This simply states that the proportion of available consults allocated to referrals received from the central intake clinic is given by the quotient of throughput acquired through central intake clinic and throughput acquired from direct referrals. The existence of an evolutionary equilibrium means that the intervention can be sustained long term (from the point of view of our evolutionary dynamic), However, it does not guarantee that the intervention is effective in its sustained state. The expected equilibrium specialist participation rate as a function of the of the proportion of referrals that are sent to the central intake clinic. If the proportion of a specialist's throughput that is acquired through central intake is sufficiently high, then full participation, $x^* = 1$, can be maintained, allowing optimal system performance to be achieved. However, if specialists are able to maintain a sufficient level of throughput from direct referrals relative to that generated from the central intake clinic, then x^* may be low at equilibrium. In such cases, the wait

time benefits generated by the central intake clinic may be small and may not be sufficient to justify the continued maintenance of the central intake clinic.

Summary

Our model of the ecological dynamics of the system demonstrated that the introduction of referral to next available specialist through an optional centralized screening clinic can produce shorter wait times between referral and specialist consult. However, it also revealed that the effectiveness and sustainability of the intervention is linked to the adaptive behaviour of specialists. The inclusion of an evolutionary dynamic to model the adaptive behaviour of specialists allows us to assess whether the intervention remains effective over the long term. In doing so, we observe that there is a risk that the effectiveness of the intervention is not sustainable. This occurs when the objectives of the specialists are at odds with what is best for the system. In minimizing wait times, the system prefers to distribute workload evenly amongst the specialists, but an even share of referrals might be less than what each specialist desires. In such cases, if a specialist can acquire a bigger share through direct referrals then the specialist will do exactly that. However, if the system generates enough referrals to the central intake clinic, then specialists will prefer to increase their allocation of consults to the central intake clinic for scheduling.
